# Indicator-based public health monitoring in old age in OECD member countries: a scoping review

**DOI:** 10.1186/s12889-019-7287-y

**Published:** 2019-08-07

**Authors:** Maike Miriam Grube, Ralph Möhler, Judith Fuchs, Beate Gaertner, Christa Scheidt-Nave

**Affiliations:** 1 Department of Epidemiology and Health Monitoring, Robert Koch Institute, Berlin, Germany; 2 Institute for Evidence in Medicine, Medical Center and Faculty of Medicine, University of Freiburg, Freiburg, Germany

**Keywords:** Public health, Monitoring, Old age, Indicators

## Abstract

**Background:**

The scoping review was conducted as part of the project “Improving Health Monitoring in Old Age” (IMOA) which aims at developing a conceptual framework with key indicators for a public health monitoring of the population aged 65 years and older in Germany. We systematically identified indicator-based monitoring systems that focus on health and wellbeing of older populations in member countries of the Organisation for Economic Co-operation and Development (OECD) and analysed them with regard to structure, development and content.

**Methods:**

A systematic search strategy included searching the websites of national public health institutes, an additional internet search and a MEDLINE search via PubMed. Indicator systems were included if they presented data on a national level, if they were published or updated after 01/01/2007, if they relied on more than one data source and if they were available in English or German. Data on the structure and development of the indicator sets were extracted using a standardized documentation form, and a content analysis of the indicators was conducted using a pre-defined conceptual framework with three health areas and 11 health domains that is based on the Worlds Health Organization’s “World Report on Ageing and Health” and on the International Classification of Functioning, Disability and Health (ICF).

**Results:**

Ten indicator-based monitoring systems met our inclusion criteria. Of these, six systems focused exclusively on older populations, and four offer a specific subset of indicators for older age. The number of indicators varied between 22 and 53 (median 32.5). Four systems were directly related to national public health or healthy ageing strategies, and two systems had been developed in consensus processes involving multiple stakeholders. The highest numbers of indicators could be assigned to the domains “health care”, “nursing and community care”, “wealth and poverty” and “physical health”. Overall, 47 different concepts could be identified in the monitoring systems.

**Conclusion:**

Among indicator-based monitoring systems of health in older age identified in member countries of the OECD, there is considerable variation with regard to structure, development and content. The results will inspire the development of a public health monitoring of the older population in Germany.

**Electronic supplementary material:**

The online version of this article (10.1186/s12889-019-7287-y) contains supplementary material, which is available to authorized users.

## Background

The proportion of older people in Germany as in other high-income countries has been increasing steadily over the past decades. In Germany, 20% of the population are 65 years and older, population forecasts predict that in 2030 28% of the population will be 65 years and older [1]. The World Health Organization’s (WHO) “World Report on Ageing and Health” [2] states that improving measurement, monitoring and understanding of health in older populations is crucial to allow targeted public health action on ageing, to optimize functional ability of older adults, to align health systems to the needs of older adults, to build sustainable long-term care systems and to create age-friendly environments. However, data on the health status and the health needs of older adults are widely insufficient and there is no systematic collection, analysis and interpretation of health data and a lack of scientific consensus on health concepts and key indicators that would be essential to the planning and evaluation of health policies and public health interventions for older adults. This is partly due to the fact that general population surveys commonly apply age limits of 80 or 85 years for study participation [3, 4] and exclude residents of non-private households from their target populations [5]. In addition, both sampling methods and data collection procedures applied in surveys often seem less adequate to reach older adults with poor health or functional limitations and those living in institutional care. However, tackling the major knowledge gaps in the health and wellbeing of older adults is of utmost importance both in Germany and in other countries worldwide.

**Fig. 1:**
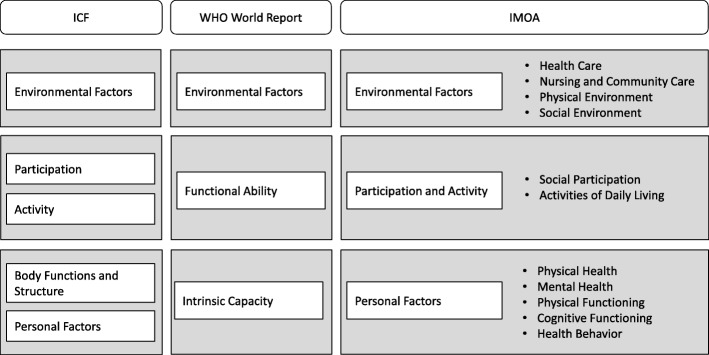
Health areas and domains of the “IMOA – Improving Health Monitoring in Old Age” project

The present study was conducted as part of a two-year research project “IMOA – Improving Health Monitoring in Old Age” funded by the Robert Bosch Stiftung. The main objective of the IMOA project was to adapt the sampling and recruitment strategies of general health surveys according to the needs and capabilities of older adults and to develop a conceptual framework with key indicators for a public health monitoring of the population aged 65 years and older. Aiming to allow general health surveys to become more inclusive of older adults, the effects of a sequential mixed-mode design including different contact and data collection modes like for example home visits and proxy interviews were tested in a pilot study in 2017–2018. Background, methods and results are described in detail elsewhere [6]. The development of a conceptual framework and definition of key indicators took place in a structured multi-level consensus process involving a broad range of researchers and practitioners [7]. Based on the WHO‘s “World Report on Ageing and Health” [2] and on the International Classification of Functioning, Disability and Health (ICF) [8], we initially defined three health areas that should constitute an overarching frame for a future public health monitoring of older adults in Germany: environmental factors, activity and participation and personal factors. Informed by a qualitative content analysis of national and international health targets on health in older age, as well as extensive consultations with experts from various fields (public health, nursing care, and geriatrics), we further agreed upon a set of 11 relevant health domains that should be covered by the indicators. This first step of the consensus process has been described previously [9] and the framework, with its health areas and domains, is illustrated in Fig. 1.

We conducted this scoping review in support of the second step of the consensus process, i.e. the selection of key indicators for a public health monitoring of the population 65 years and older in Germany. The purpose of the review was to systematically identify indicator-based approaches to monitor the health and wellbeing of older populations in countries of the Organisation for Economic Co-operation and Development (OECD) and to analyze the structure, development and the content of the systems in order to answer the following questions: (1) What are the main characteristics of indicators-based monitoring systems on health in older age, and to what extent do monitoring systems differ? (2) Have the indicator sets been developed in structured consensus processes involving multiple stakeholders and are they used to monitor the implementation and success of public health or healthy ageing strategies? (3) Which health domains and concepts are covered by the indicator sets?

## Methods

The review follows an approach suggested by Arkey and O’Malley [10], who describe different stages for conducting a scoping review: (1) identifying the research questions, (2) identifying relevant studies, (3) study selection, and (4) charting the data, collating, summarizing and reporting the results.

### Identifying relevant studies

A comprehensive search strategy was used to identify indicator-based approaches to monitor the health status of older populations.

### Data sources

We searched the websites of public health institutes and conducted an additional key word search using the internet search engine Google for the 35 member countries of the OECD. Websites of public health institutes were identified using the openly accessible member list of the “International Association of National Public Health Institutes” (www.ianphi.org), for some countries not included in this list additional internet searches helped to identify websites of national public health institutes. We further searched MEDLINE (via PubMed) for relevant articles describing indicator sets in any of the OECD member countries. The search was conducted in June–July 2017.

### Key words for internet and electronic database search

The search string included a combination of key words as follows:

#### Websites

Indicators; ageing/aging; old age; older persons.

#### Google

Healthy ageing/aging; positive ageing; health in old age; health of older persons; indicators; monitoring; surveillance.

#### MEDLINE (via PubMed)

(health status indicators[MeSH] OR public health surveillance[MeSH] OR health monitor*[TIAB]) AND (indicator[TIAB]) AND (aged[MeSH] OR elderly[TIAB] OR aged[TIAB])

### Inclusion criteria

We included all types of published information on indicators used to monitor health or wellbeing in older age on a national level^1^ in member states of the OECD, websites, brochures, reports and scientific papers. We included documents that met the following inclusion criteria:

1. Indicators were available in English or German language
2. Indicator sets relied on more than one data source, e.g. surveys and registers
3. Information on how the indicators are defined was provided
4. Indicators were published or updated between 01/01/2007 and 31/07/2017

### Study selection

**Fig. 2:**
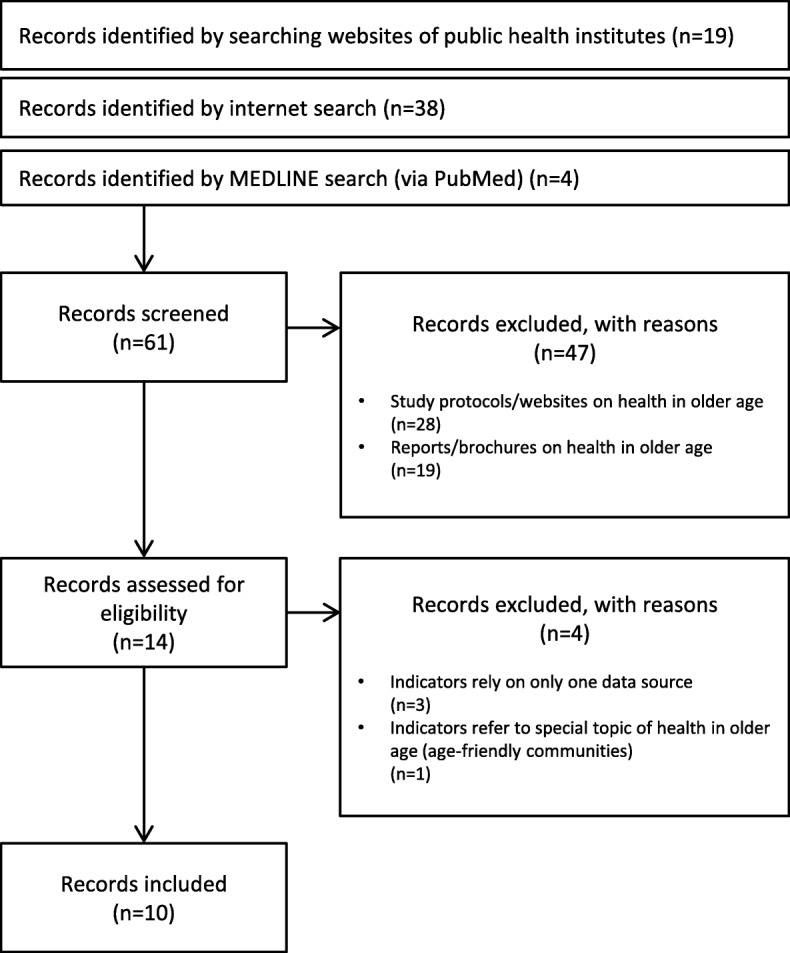
Selection process

In the first step, we identified 61 documents (Fig. 2). The majority of documents were identified by searching the websites of public health institutes (*n* = 19) and by searching the internet using Google (*n* = 38). In addition, four documents could be identified via PubMed. Of the 61 documents screened, 47 documents were excluded because they did not provide indicator-based information on the health status of older populations. Of the documents excluded, 28 documents referred to surveys on health in older age instead and had heterogeneous formats, such as study protocols, study websites, technical reports or research articles (Australia (*n* = 1), Austria (*n* = 1), Belgium (*n* = 1), Canada (*n* = 1), Chile (*n* = 1), Denmark (*n* = 1), England (*n* = 1), Finland (*n* = 1), Germany (*n* = 1), Iceland (*n* = 1), Ireland (*n* = 1), Israel (*n* = 1), Italy (*n* = 1), Japan (*n* = 2), Korea (*n* = 2), Mexico (*n* = 1), Netherlands (*n* = 1), New Zealand (*n* = 1), Norway (*n* = 1), Poland (*n* = 1), Portugal (*n* = 1), Spain (*n* = 1), Sweden (*n* = 2), USA (*n* = 2)). A total of 19 documents provided information on health in older age on a national level but did not refer to any pre-defined indicators and were thus excluded (Australia (*n* = 2), Austria (*n* = 1), Canada (*n* = 1), France (*n* = 3), Germany (*n* = 2), Netherlands (*n* = 1), Norway (*n* = 2), Scotland (*n* = 2), Slovenia (*n* = 1), Switzerland (*n* = 2), Wales (*n* = 2)). The remaining 14 documents, which provided indicator-based information on health in older age, were assessed in full text for eligibility. Three documents were further excluded because the indicators presented relied on data from a single survey (Canada (*n* = 1), Turkey (*n* = 1), United States of America (*n* = 1)). A fourth document was excluded because the indicator set referred to a special aspect of health in older age, namely, to age-friendly communities, and was assessed not on a population level, but on a community level (Canada (*n* = 1)). Finally, a total of ten documents were included in the review (Finland (*n* = 2), Ireland (*n* = 1), New Zealand (*n* = 2), Switzerland (*n* = 1), the United Kingdom (England (*n* = 1), Scotland (*n* = 1), Wales (*n* = 1)), and the United States of America (*n* = 1)) [11–19]. Excluded documents are listed in Additional file 1.

### Charting the data, collating, summarizing and reporting the results

Data on structure and development of the indicator sets were extracted using a standardized documentation form. We extracted information on the number of indicators, publishing institution, last update, data sources the indicators relied on, graphic presentation of indicators and metadata on the indicators such as rationale or data restrictions that were provided. Information on how the indicators were developed and on whether they were based on public health frameworks or healthy ageing strategies was summarized in brief. Data were extracted by one reviewer (MG) and a second reviewer (BG) performed an independent data extraction for a randomly chosen subset of three monitoring systems. Inconsistent results were discussed and resolved between both reviewers.

To analyse the content of the monitoring systems, the indicators were assigned to one of the 11 health domains within the pre-defined conceptual framework of the “IMOA – Improving Health Monitoring in Old Age” project:

#### Environmental factors

1. Health care
2. Nursing and community care
3. Physical environment
4. Social environment

#### Activities and participation

1. Social participation
2. Activities of daily living

#### Personal factors

1. Physical health
2. Mental health
3. Physical functioning
4. Cognitive functioning
5. Health behaviour

Since a large number of indicators could not be assigned to any of the pre-defined health domains, the following three ancillary domains “demographics”, “life expectancy/mortality” and “wealth/poverty”, were added in an inductive approach and summarized as “context factors”. In a next step, the indicators were clustered into different concepts within the 11 health domains and the three additional domains. We assigned indicators to concepts if at least two indicators from different monitoring systems or at least three indicators from the same monitoring system were of related content.

## Results

### Structure of the monitoring systems

Indicator-based monitoring activities on health in older age could be identified for Finland (*n* = 2), Ireland (*n* = 1), New Zealand (*n* = 2), Switzerland (*n* = 1), the United Kingdom (England (*n* = 1), Scotland (*n* = 1), Wales (*n* = 1)), and the United States of America (*n* = 1) (Fig. 2).

A short overview of the main characteristics of the monitoring systems is presented in Table 1. Six monitoring systems focused exclusively on health and wellbeing in older age [12–17], whilst four systems had a broader focus on health and wellbeing in all age groups but offered a specific subset of indicators for health and wellbeing in older age [11, 18–20]. Most of the monitoring activities included in the review were provided by national public health institutes or public health observatories; however, some had been published by health or social ministries or other agencies [12, 14, 16, 21]. Six monitoring systems provided websites or online tools to present indicator-based information on health in older age [11, 13–15, 18, 20]. Three monitoring systems provided indicator-based health information in periodically published reports [12, 16, 17]. One monitoring system had not yet been implemented [19]. The number of indicators for health and wellbeing in older age in the included monitoring systems varied between 22 and 53 (median 32.5) in the indicator systems focusing on the older population and between 5 and 63 (median 11) in the age-related subsets included in broader indicator systems. Most but not all indicator systems provided data on the indicators disaggregated by sex and age groups. Some systems also provided information for different education or income groups or disaggregated by area deprivation level, rurality or ethnicity. Three monitoring systems displayed indicators only on the national level [12, 14, 16], whilst six monitoring systems displayed their data on one or more regional levels and thus offered a regional comparison [11, 13, 15, 18, 20, 21]. Three of the monitoring systems also offered a graphical visualization of regional data using maps of different scales [11, 13, 18]. Three of the monitoring systems included in the review provided online-based area profiles that offered detailed cross-indicator information for any selected region, comparing regional values with values for the whole country and displaying the inter-regional variation [13, 15, 20]. Certain metadata on the indicators were provided by all the monitoring systems included in the analysis: A detailed definition of indicators and data sources were made available for all the indicator sets. Five systems published at least a short rationale for the indicators chosen [11, 13, 16, 17, 19], three systems included information on possible data restrictions [17, 18, 20], and two systems included interpretation guidance [18, 20]. One system included advice for local authorities on how to act on specific issues [20].

**Table 1:** Characteristics of the indicator-based monitoring systems

Finland - Finnish Welfare Compass [20]	
Publishing institution	National Institute for Health and Welfare (THL)
No. of indicators	16
Reference population	0+ years
Last update	2017
Website or report/brochure	Website
Geographical levels	National level and different regional levels
Disaggregation levels	Sex
Development of the indicators	Structured consensus process involving multiple stakeholders
Graphic display	Line charts displaying the indicators over time; area profiles with detailed information for any selected region (spine charts comparing values for a selected region with values for the whole country and displaying the region with the lowest and highest value as well as interquartile ranges)
Metadata	Definition, data sources, items/instruments, available time series, data restrictions, interpretation guidance, impact on population wellbeing, financial impact, advice for local authorities
Finland - Statistical information on welfare and health in Finland (SOTKAnet) [18]	
Publishing institution	National Institute for Health and Welfare Finland (THL)
No. of indicators	57
Reference population	0+ years
Last update	2017
Website or report/brochure	Website
Geographical levels	National level and different regional levels
Disaggregation levels	Sex
Development of the indicators	No information available
Graphic display	No default settings; users are free to create a broad range of tables, line and bar charts including geographical comparisons and time series, as well as maps displaying the indicators
Metadata	Definition, data sources, disaggregation levels, data restrictions, interpretation guidance, legislation
Ireland - Positive Ageing 2016. National indicators report [16]	
Publishing institution	Healthy and Positive Ageing Initiative (HaPAI), Department of Health
No. of indicators	53
Reference population	50+ years
Last update	2016
Website or report/brochure	Report/brochure
Geographical levels	National level
Disaggregation levels	Sex, age group
Development of the indicators	Based on the Irish National Positive Ageing Strategy; structured consensus process involving multiple stakeholders
Graphic display	Line and bar charts displaying the indicators over time
Metadata	Definition, data sources, rationale, literature (for some indicators), for data sources: reference period, data collection frequency, coverage, method of data collection, data content, relevant policy areas, references, sample size
New Zealand - Positive Aging Indicators [17]	
Publishing institution	Ministry of Social Development
No. of indicators	34
Reference population	65+ years
Last update	2007
Website or report/brochure	Report/brochure
Geographical levels	National level and regional level (some indicators)
Disaggregation levels	Sex, age group, ethnicity; some indicators: area deprivation levels, personal income, education, living arrangements, urban/rural
Development of the indicators	Based on the New Zealand Positive Ageing Strategy
Graphic display	Line and bar charts displaying the indicators over time
Metadata	Definition, data sources, items/instruments, data restrictions, reference population, rationale
New Zealand - Older people’s health data and stats [14]	
Publishing institution	Ministry of Health
No. of indicators	22
Reference population	15+ years
Last update	2016
Website or report/brochure	Website
Geographical levels	National level
Disaggregation levels	Sex (some indicators), age group, Maori and non-Maori population
Development of the indicators	No information available
Graphic display	Line and bar charts displaying the indicators over time
Metadata	Definition, data sources
Switzerland - Indicators for health in old age [11]	
Publishing institution	Swiss health observatory
No. of indicators	7
Reference population	15+ years
Last update	2016
Website or report/brochure	Website
Geographical levels	National level and regional level
Disaggregation levels	Sex, age group, speech area, education, income, urban/rural
Development of the indicators	No information available
Graphic display	Line and bar charts displaying the indicators over time; ranked bar charts with indicator values and 95% confidence intervals for regions; crude numbers of persons affected in a region; maps offer a regional comparison of indicators; most data are also available as Excel files
Metadata	Definition, data sources, items/instruments, rationale
England (UK) - Older People’s Health and Wellbeing [13]	
Publishing institution	Public Health England
No. of indicators	31
Reference population	65+ years
Last update	2017
Website or report/brochure	Website
Geographical levels	Whole country and different regional levels
Disaggregation levels	Sex (some indicators)
Development of the indicators	Based on the Public Health Outcomes Framework for England, the Adult Social Care Outcomes Framework and the National Health Service Outcomes Framework
Graphic display	Line charts displaying the indicators over time; area profiles with detailed information for any selected region (spine charts comparing values for a selected region with the English average or any other comparator and displaying the region with the highest and lowest value as well as interquartile ranges); ranked bar charts with values and 95% confidence intervals for regions comparing them either with the English average or with pre-defined target values using a modified traffic-light system; crude numbers of persons affected in a region; maps displaying indicators in a traffic-light system against the benchmark, or in a continuous, quartile-based or quintile-based colour scheme; most data are also available as Excel files
Metadata	Definition, data sources, items/instruments, reference population, rationale
Scotland (UK) - Online Profiles: Older People 65+ [15]	
Publishing institution	Scottish Public Health Observatory
No. of indicators	26
Reference population	65+ years
Last update	2014
Website or report/brochure	Website
Geographical levels	Two different regional levels
Disaggregation levels	–
Development of the indicators	No information available
Graphic display	Area profiles with detailed information for any selected region (spine charts comparing values for a selected region with the Scottish average or any other comparator and displaying the region with the highest and lowest value as well as interquartile ranges); spine charts show the crude number of persons affected in a region; a colour-coded modified traffic-light system is used to indicate statistical significance of the deviation of regional values from the Scottish average; benchmark comparison of regions with the Scottish average and with other regions using bar charts that include 95% confidence intervals
Metadata	Definition, data sources
Wales (UK) - Measuring the health and well-being of a nation: Public Health Outcomes Framework for Wales; Healthy Ageing [19]	
Publishing institution	Public Health Wales (not yet implemented)
No. of indicators	5
Reference population	0+ years
Last update	Not yet implemented
Website or report/brochure	–
Geographical levels	To be presented at different regional levels
Disaggregation levels	Sex, age group, urban/rural
Development of the indicators	Based on the Public Health Outcomes Framework for Wales
Graphic display	–
Metadata	Definition, data sources, rationale
USA - Older Americans: Key Indicators of Well-Being [12]	
Publishing institution	Federal Interagency Forum on Aging-Related Statistics
No. of indicators	41
Reference population	65+ years
Last update	2016
Website or report/brochure	Report/ brochure
Geographical levels	National level
Disaggregation levels	Sex (some indicators), age group, ethnicity
Development of the indicators	No information available
Graphic display	Bar and line charts displaying the indicators over time
Metadata	Definition, data sources, reference population

### Development of the monitoring systems

Four of the ten monitoring systems included in the analysis were directly related to national public health frameworks or healthy ageing strategies: The indicators of the Irish Healthy and Positive Ageing Initiative (HaPAI) [16], the New Zealand Positive Ageing Indicators [17], the indicators presented in the English Older People’s Health and Wellbeing Profile [13] and the indicators presented in the Public Health Outcomes Framework for Wales [19].

The Irish HaPAI indicators [16] were developed in order to monitor and evaluate progress of the Irish National Positive Ageing Strategy [22], which had been published by the Department of Health in 2013 after a structured consensus process that included a public call for written submissions, a series of public regional consultation meetings and meetings with groups representing vulnerable and marginalized older people. The New Zealand Positive Ageing Indicators [17] were based on the principles and goals of the New Zealand Positive Ageing Strategy, which had been released by the Ministry of Social Development in 2001 after an extensive consultation process that included focus groups with a broad range of stakeholders [21]. However, the indicators have not been presented as a tool to evaluate the future progress of the Positive Ageing Strategy. For this purpose, a two-tier monitoring system based on short annual reports and less frequently published comprehensive reports had been suggested instead [21]. The English Older People’s Health and Wellbeing Profile [13] belonged to a number of public health profiles developed by Public Health England that provided indicator-based information on population health for different population groups and various sub-topics (e.g., oral health, diabetes). The majority of the indicators presented in the online tool originated from the Public Health Outcomes Framework for England, which was developed in a broad public consultation process and has subsequently been updated on a constant basis [23]. Some indicators presented in the Older People’s Health and Wellbeing Profile originated from the Adult Social Care Outcomes Framework and the National Health Service outcomes framework, which are closely linked to the Public Health Outcomes Framework [24]. The indicators presented in the Public Health Outcomes Framework for Wales were tailored to the health targets outlined in the framework and were meant to assess future progress in improving population health. Publication of the Public Health Outcomes Framework for Wales followed an extensive consultation process in which approximately 60 organizations from the voluntary sector, health boards, housing associations, pharmacies, local government, as well as members of the public, took part [19].

Two of the indicator sets included in the analysis were developed in a structured consensus process involving multiple stakeholders: The indicators of the Irish Healthy and Positive Ageing Initiative (HaPAI) [16] and the key indicators of the Finnish National Institute for Health and Welfare (THL) [20]. The Irish indicators [16] were developed based on the Irish National Positive Ageing Strategy [22]. In addition, the development of indicators was informed by an extensive literature review on key areas that impact positive ageing and that had been outlined in the National Positive Ageing Strategy. The subsequent consensus process included an adapted Delphi technique that invited members from research and academic networks, networks of older people, local authorities and government departments [16]. The Finnish THL key indicators [20] were developed in a consensus process initiated and coordinated by the National Institute for Health and Welfare (THL) that included a web-based survey and consultations with multiple stakeholders, such as representatives of ministries, local authorities, universities, research institutes, statistical offices and social insurances between 2009 and 2012.

### Content of the monitoring systems

**Fig. 3:**
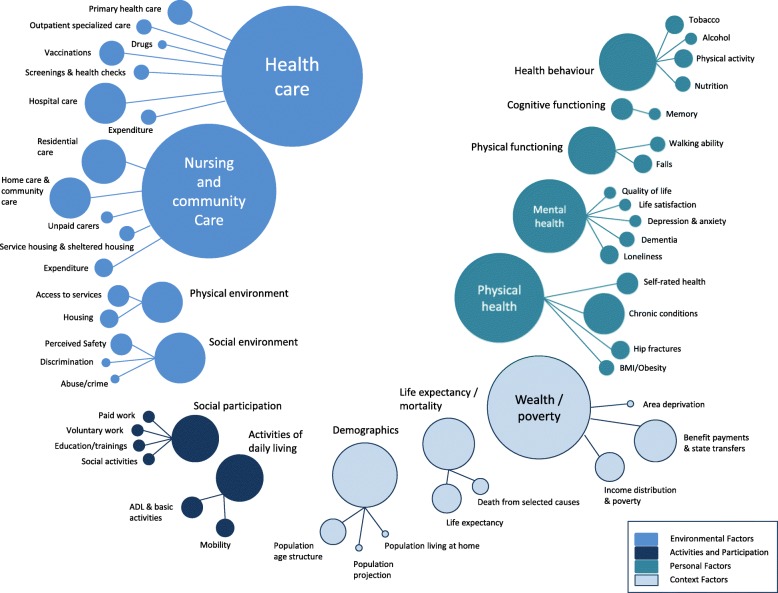
Health areas, domains and concepts covered by the indicators in the monitoring systems included. Indicators that could not be clustered into concepts due to their unique content are listed below:Health care: share of health services used by people aged 65 and over; Medicare-covered physician and home health care visits; veterans’ health care. Nursing and community care: adequate assistance, caregiver stress, comprehensive clinical assessments. Physical environment: outdoor air quality. Social environment: social support; local area social capital; trust in institutions; trust in others; community inclusion. Social participation: caring for relatives and children; participation in cultural and arts activities. Activities of daily living: deterioration of work ability; internet use; time spent doing selected activities. Mental health: severe mental strain; sense of control; positive perceptions of own age; suicides. Cognitive functioning: literacy skill; numeracy skills; mild cognitive impairment. Demographics: living alone; engagement with te ao Māori; te reo Māori speakers; racial and ethnic composition; marital status; educational attainment; living arrangements; older veterans. Life expectancy/mortality: deaths in usual place of residence; excess winter deaths. Wealth/poverty: recipients of full national pension; living standards involving some degree of hardship; home ownership; housing affordability; fuel poverty; winter fuel payments; household net worth; total household annual expenditures

The ten monitoring systems included in this review contained 293 indicators. The distribution of indicators among domains and concepts is displayed in Fig. 3. A total of 111 indicators were assigned to the health area “environmental factors”, 28 indicators were assigned to “activities and participation”, 86 indicators were assigned to “personal factors” and 68 indicators were assigned to the additional category “context factors”. Within the 14 domains, most indicators were assigned to the domains “health care” (43 indicators) and “nursing and community care” (41 indicators). The domains “wealth and poverty” (32 indicators) and “physical health” (27 indicators) have also been assigned a large number of indicators. We further clustered the indicators into 47 different concepts within the 14 domains. Forty-eight of the 293 indicators could not be categorized into concepts due to their unique content and were categorized under the subheading “other”. Concepts that included the highest numbers of indicators were “residential care” (13 indicators), “benefit payments and state transfers” (13 indicators), “hospital care” (12 indicators), “home care and community care” (12 indicators) and “chronic conditions” (12 indicators).

## Discussion

This scoping review of existing public health monitoring systems on health in older age among the 35 OECD countries was conducted to inform the selection of key indicators in order to develop an indicator-based national public health monitoring of the population 65 years or older in Germany. We identified ten indicator-based monitoring systems in a total of eight OECD countries, including Ireland, Switzerland, the United States of America, Finland and New Zealand and the UK with separate indicator sets for England, Scotland and Wales. Current indicator-based monitoring activities for health in older age proved to be very heterogeneous with regard to their structure, development and content. Some indicator sets focused exclusively on health and wellbeing in older age, whereas others referred to the whole population but defined a subset of indicators for health in older age. Not only did the number of indicators vary greatly between the monitoring systems, so too did the format used to present the indicators. Formats ranged from periodically published reports or brochures to websites or complex online tools offering a variety of functions. The majority of monitoring systems displayed the indicators not only on a national level but also on one or more regional levels, for example, for regions, districts or local authorities [11, 13, 15, 17, 18, 20] and thus addressed policy makers and health care planners both on the national level and in municipalities. Various methods of data visualization were used. We would like to specifically highlight the area profiles that were offered in some of the monitoring systems [13, 15, 20] and the geographical display of indicators using maps that was available in some of the indicator systems [11, 13, 18]. The area profiles offered detailed information for any selected region with spine charts comparing values for a region with the national average. A geographical display of selected indicators using maps allowed a quick comparison of regions. In the English example [13], a traffic-light system comparing regional values against the benchmark was available as were continuous, quartile-based or quintile-based colour schemes displaying the values on regional levels. The amount of metadata that was provided for the indicators varied significantly. Data sources used for the indicators were made available in all the monitoring systems included in the review. The majority of monitoring systems offered a detailed definition of the indicators and a short rationale for the indicators. Some monitoring systems also included interpretation guidance, information on data restrictions or advice for local authorities on how to act on specific issues.

We also analysed how the indicator sets were developed and whether they were based on a national public health framework or healthy ageing strategy. We found that four out of ten indicator sets were entirely or in parts tied to a national strategy [13, 16, 17, 19]. However, only two of these four indicator sets were developed explicitly in order to monitor the progress of the underlying strategy, whilst this was not the case for the two other sets. Two of the indicator sets were developed in extensive and long-dated public consensus processes [16, 20]. These processes included consultations with a broad range of stakeholders, such as government departments, municipalities, research institutes, academic networks, networks of older people, statistical offices, social insurances, health boards, housing associations and members of the public. Methods used to receive feedback and to form a consensus on the indicators included a public call for written submissions, a Delphi technique, a web-based survey, focus groups and bilateral as well as multilateral consultation meetings.

Regarding the content of the indicator sets, we found that the largest numbers of indicators referred to the two health domains “health care” and “nursing and community care” and to the domain “wealth or poverty”. However, in addition to these, a wide range of other concepts were covered by the indicators, which shows that the underlying definition of wellbeing and health in older age was rather broad and holistic. It was beyond the scope of this review to compare indicator sets between countries in more detail, i. e. with regard to specific health priorities, indicators and instruments applied. Future research efforts will be needed to examine the options to harmonize concepts, indicators and instruments between countries in order to permit international comparisons.

To our knowledge, indicator-based national monitoring systems that focus on health and wellbeing of older populations have not previously been reviewed in detail. As we could expect a large number of theoretical frameworks and health concepts to be covered, we chose to conduct a scoping review. A strength of this approach is that we were able to summarize and analyse a broad range of different publication types and to provide an extensive overview of national monitoring systems on health in older age. We performed a systematic search including both a structured database search and an extensive search on the websites of relevant organisations in this field. However, this review also has some limitations. One of the main limitations of our review was the restriction to documents that were available in English or German language. Websites or documents providing information on national health indicators mainly address professionals in health and social care and policy makers both on the national and regional levels, not an international scientific community. Seven out of the ten indicator sets included in the analyses originated from English-speaking countries, which is a highly selective choice of indicator sets. We can assume that more OECD countries provide relevant indicator sets that are exclusively published in the respective national languages. Secondly, additionally contacting public health authorities in OECD countries might have revealed additional information and might have guided the interpretation of information already available to us. We had considered this additional step at the beginning of our study, but decided against it, because the scoping review aimed at identifying, describing and summarizing indicators that are used for health reporting and are openly available and visible to the public. As a third limitation, we restricted our search to monitoring systems that either focused on health and wellbeing in older age or that specified a subset of indicators as relevant for health in older age. We thus excluded public health monitoring systems that referred to the whole population without defining subsets of indicators for specific age groups. Although the present study was not apt to provide an exhaustive review of international monitoring approaches in old age, its results helped to inform the development of an indicator-based public health monitoring of the older population in Germany. The indicators identified and described here were examined for duplicates and presented to an expert panel for selection of indicators in a multistage structured consensus process. Eventually, a total of 18 indicators from three health areas (environmental factors, activities and participation, and personal factors) were selected as being most relevant to establishing health monitoring for older people in Germany [7].

## Conclusion

Our scoping review illustrated that indicator-based national public health monitoring activities of older adults are highly diverse in the OECD member countries included in the analysis. A wide range of different concepts relevant to older persons’ health is covered in the monitoring systems. Some indicator sets have been developed based on existing national strategies and in elaborate consultation processes, whereas others have not or simply do not provide details on their development process. In addition, there is much variety regarding the health concepts and constructs covered as well as in the way health information is presented and indicators are displayed. While this may in part reflect country-specific differences in health care systems and health needs, further research is needed to explore the opportunities and limitations of international standards. In Germany, the results of the present study laid the ground to stimulate an expert consensus process on indicator selection for future population-based health monitoring of the population 65 years and older. For future international efforts to implement or improve monitoring systems on health in older age we highly recommend to consider the broad range of possible approaches that have been outlined in our review before deciding on a strategy which fits the particular context, needs and expectations. This includes decisions on main concepts and key indicators as well as the stakeholders involved and the formats chosen for timely and effective dissemination of results. Most importantly, specific approaches will always be guided by national health priorities and health goals as well as available data and resources.

## Additional file

Additional file 1: Documents excluded in the study selection process. (DOCX 23 kb)

## Data Availability

Not applicable

## Abbreviations

ADL: Activities of daily living
HaPAI: Irish Healthy and Positive Ageing Initiative
ICF: International Classification of Functioning, Disability and Health
IMOA: Improving Health Monitoring in Old Age
OECD: Organisation for Economic Co-operation and Development
THL: National Institute for Health and Welfare Finland
WHO: World Health Organization

## Footnotes

1 England, Scotland, Wales and Northern Ireland were included as separate entities, since they have independent public health systems.
