# Supplementary material for: Indicator-based public health monitoring in old age in OECD member countries: a scoping review
Source: BMC Public Health. 2019 Aug 7;19:1068. doi: 10.1186/s12889-019-7287-y (PMC6686554; doi:10.1186/s12889-019-7287-y)
Supplement: Supplementary file 1 — Documents excluded in the study selection process. (DOCX 23 kb) [file 12889_2019_7287_MOESM1_ESM.docx]

**Additional file 1**

**Documents excluded in the study selection process**

1. Documents referring to surveys on health in older age (n=28)

**Australia (n=1)**

Survey of Disability, Ageing and Carers, Summary of Findings 2015. Australian Bureau of Statistics.
http://www.abs.gov.au/ausstats/abs@.nsf/mf/4430.0 (study website)

**Austria (n=1)**

Österreichische Interdisziplinäre Hochaltrigenstudie . Zusammenwirken von Gesundheit, Lebensgestaltung und Betreuung. Austrian Interdisciplinary Platform on Ageing (ÖPIA). https://www.sozialministerium.at/cms/site/attachments/6/9/5/CH3434/CMS1460108200204/oeihs_i_endbericht_endfassung.27.4.2015.pdf (report)

**Belgium (n=1)**

Belgian Ageing Studies (BAS). Free University of Brussels and University College Ghent.
http://www.belgianageingstudies.be (study website)

**Canada (n=1)**

Canadian Longitudinal Study on Aging (CLSA). CLSA national research collaboration.
https://www.clsa-elcv.ca (study website)

**Chile (n=1)**

National Survey of Dependency (NSD) in the elderly.
[Fuentes](https://www.ncbi.nlm.nih.gov/pubmed/?term=Fuentes%20P%5BAuthor%5D&cauthor=true&cauthor_uid=29213920)^,^ P, Albala C. An update on aging and dementia in Chile. Dement Neuropsychol. 2014; 8(4): 317–22 (research article)

**Denmark (n=1)**

The Danish Longitudinal Study of Ageing. The Danish National Centre for Social Research.
http://cssr.surveybank.aau.dk/webview/index/en/MyServer/aeldredatabasen.d.23/aeldredatabasen/fStudy/TheDanishLongitudinalStudyofAgeing (technical report)

**England (n=1)**

English Longitudinal Study of Ageing (ELSA). University College London (UCL), Institute for Fiscal Studies, NatCen Social Research, University of Manchester
https://www.elsa-project.ac.uk. (study website)

**Finland (n=1)**

Health Behaviour and Health among the Finnish Retirement-Age Population (EVTK). National Institute for Health and Welfare.
https://thl.fi/en/web/thlfi-en/research-and-expertwork/population-studies (study website)

**Germany (n=1)**

Deutscher Alterssurvey (DEAS): Die zweite Lebenshälfte. Deutsches Zentrum für Altersfragen.
https://www.dza.de/forschung/deas.html (study website)

**Iceland (n=1)**

Ages Reykjavik Study.
[Fisher D](https://www.ncbi.nlm.nih.gov/pubmed/?term=Fisher%20D%5BAuthor%5D&cauthor=true&cauthor_uid=23996030), [Li CM](https://www.ncbi.nlm.nih.gov/pubmed/?term=Li%20CM%5BAuthor%5D&cauthor=true&cauthor_uid=23996030), [Chiu MS](https://www.ncbi.nlm.nih.gov/pubmed/?term=Chiu%20MS%5BAuthor%5D&cauthor=true&cauthor_uid=23996030), [Themann CL](https://www.ncbi.nlm.nih.gov/pubmed/?term=Themann%20CL%5BAuthor%5D&cauthor=true&cauthor_uid=23996030), [Petersen H](https://www.ncbi.nlm.nih.gov/pubmed/?term=Petersen%20H%5BAuthor%5D&cauthor=true&cauthor_uid=23996030), [Jónasson F](https://www.ncbi.nlm.nih.gov/pubmed/?term=J%C3%B3nasson%20F%5BAuthor%5D&cauthor=true&cauthor_uid=23996030), [Jónsson PV](https://www.ncbi.nlm.nih.gov/pubmed/?term=J%C3%B3nsson%20PV%5BAuthor%5D&cauthor=true&cauthor_uid=23996030), [Sverrisdottir JE](https://www.ncbi.nlm.nih.gov/pubmed/?term=Sverrisdottir%20JE%5BAuthor%5D&cauthor=true&cauthor_uid=23996030), [Garcia M](https://www.ncbi.nlm.nih.gov/pubmed/?term=Garcia%20M%5BAuthor%5D&cauthor=true&cauthor_uid=23996030), [Harris TB](https://www.ncbi.nlm.nih.gov/pubmed/?term=Harris%20TB%5BAuthor%5D&cauthor=true&cauthor_uid=23996030) et al. Impairments in hearing and vision impact on mortality in older people: the AGES-Reykjavik Study. Age Ageing. 2014;43(1):69-76 (research article)

**Ireland (n=1)**

Irish Longitudinal Study on Ageing (TILDA). Trinity College, Dublin.
https://tilda.tcd.ie (study website)

**Israel (n=1)**

Mabat-Zahav Israeli National Health and Nutrition Survey Ages 65 and over. Israel Center for Disease Control and Ministry of Health.
https://www.health.gov.il/UnitsOffice/ICDC/mabat/Documents/Background_English_Mabat_Zahav.pdf (technical report)

**Italy (n=1)**

The Italian Longitudinal Study on Aging (ILSA).
Maggi S, [Zucchetto M](https://www.ncbi.nlm.nih.gov/pubmed/?term=Zucchetto%20M%5BAuthor%5D&cauthor=true&cauthor_uid=7748921), [Grigoletto F](https://www.ncbi.nlm.nih.gov/pubmed/?term=Grigoletto%20F%5BAuthor%5D&cauthor=true&cauthor_uid=7748921), [Baldereschi M](https://www.ncbi.nlm.nih.gov/pubmed/?term=Baldereschi%20M%5BAuthor%5D&cauthor=true&cauthor_uid=7748921), [Candelise L](https://www.ncbi.nlm.nih.gov/pubmed/?term=Candelise%20L%5BAuthor%5D&cauthor=true&cauthor_uid=7748921), [Scarpini E](https://www.ncbi.nlm.nih.gov/pubmed/?term=Scarpini%20E%5BAuthor%5D&cauthor=true&cauthor_uid=7748921), [Scarlato G](https://www.ncbi.nlm.nih.gov/pubmed/?term=Scarlato%20G%5BAuthor%5D&cauthor=true&cauthor_uid=7748921), [Amaducci L](https://www.ncbi.nlm.nih.gov/pubmed/?term=Amaducci%20L%5BAuthor%5D&cauthor=true&cauthor_uid=7748921). The Italian Longitudinal Study on Aging (ILSA): design and methods. Aging (Milano). 1994;6(6):464-73 (research article)

**Japan (n=2)**

The Nihon University Japanese Longitudinal Study of Aging (NUJLSOA).
https://iafor.org/the-nihon-university-japanese-longitudinal-study-of-aging-nujlsoa-history-and-new-directions-yasuhiko-saito (study website)

Japanese Study of Aging and Retirement (JSTAR). Research Institute of Economy, Trade and Industry (RIETI), Hitotsubashi University, University of Tokyo.
https://www.rieti.go.jp/en/projects/jstar (study website)

**Korea (n=2)**

The Korean urban rural elderly cohort study (KURE).
Lee EY, [Kim HC](https://www.ncbi.nlm.nih.gov/pubmed/?term=Kim%20HC%5BAuthor%5D&cauthor=true&cauthor_uid=24641351), [Rhee Y](https://www.ncbi.nlm.nih.gov/pubmed/?term=Rhee%20Y%5BAuthor%5D&cauthor=true&cauthor_uid=24641351), [Youm Y](https://www.ncbi.nlm.nih.gov/pubmed/?term=Youm%20Y%5BAuthor%5D&cauthor=true&cauthor_uid=24641351), [Kim KM](https://www.ncbi.nlm.nih.gov/pubmed/?term=Kim%20KM%5BAuthor%5D&cauthor=true&cauthor_uid=24641351), [Lee JM](https://www.ncbi.nlm.nih.gov/pubmed/?term=Lee%20JM%5BAuthor%5D&cauthor=true&cauthor_uid=24641351), [Choi DP](https://www.ncbi.nlm.nih.gov/pubmed/?term=Choi%20DP%5BAuthor%5D&cauthor=true&cauthor_uid=24641351), [Yun YM](https://www.ncbi.nlm.nih.gov/pubmed/?term=Yun%20YM%5BAuthor%5D&cauthor=true&cauthor_uid=24641351), [Kim CO](https://www.ncbi.nlm.nih.gov/pubmed/?term=Kim%20CO%5BAuthor%5D&cauthor=true&cauthor_uid=24641351). The Korean urban rural elderly cohort study: study design and protocol. BMC Geriatr. 2014; 14: 33; doi: 10.1186/1471-2318-14-33 (research article)

Korean Longitudinal Study of Aging (KLoSA). Korea Employment Information Service (KEIS).
Jang SN. Korean Longitudinal Study of Ageing (KLoSA): overview of research design and contents. In: Pachana N, editor. Encyclopedia of geropsychology. Singapore: Springer; 2016 (book chapter)

**Mexico (n=1)**

Mexican Health and Aging Study (MHAS). University of Texas Medical Branch (UTMB), Instituto Nacional de Estadística y Geografía (INEGI, Mexico), University of Wisconsin, Instituto Nacional de Geriatría (INGER, Mexico), and the Instituto Nacional de Salud Pública (INSP, Mexico).
http://www.mhasweb.org (study website)

**Netherlands (n=1)**

Longitudinal Aging Study Amsterdam (LASA). VU University and VU University Medical Center Amsterdam, Netherlands and Ministry of Health, Welfare and Sports, Netherlands.
https://www.lasa-vu.nl/index.htm (study website)

**New Zealand (n=1)**

New Zealand Survey of Older People in 2000. Statistics New Zealand.
https://www.msd.govt.nz/documents/about-msd-and-our-work/publications-resources/monitoring/livingappendices.pdf (technical report)

**Norway (n=1)**

Norwegian panel study on life course, ageing and generation (NorLAG). Norwegian Social Research (NOVA) and Staistics Norway.
https://blogg.hioa.no/norlag?lang=en (study website)

**Poland (n=1)**

PolSenior Project.
Bledowski P, [Mossakowska M](https://www.ncbi.nlm.nih.gov/pubmed/?term=Mossakowska%20M%5BAuthor%5D&cauthor=true&cauthor_uid=21979452), [Chudek J](https://www.ncbi.nlm.nih.gov/pubmed/?term=Chudek%20J%5BAuthor%5D&cauthor=true&cauthor_uid=21979452), [Grodzicki T](https://www.ncbi.nlm.nih.gov/pubmed/?term=Grodzicki%20T%5BAuthor%5D&cauthor=true&cauthor_uid=21979452), [Milewicz A](https://www.ncbi.nlm.nih.gov/pubmed/?term=Milewicz%20A%5BAuthor%5D&cauthor=true&cauthor_uid=21979452), [Szybalska A](https://www.ncbi.nlm.nih.gov/pubmed/?term=Szybalska%20A%5BAuthor%5D&cauthor=true&cauthor_uid=21979452), [Wieczorowska-Tobis K](https://www.ncbi.nlm.nih.gov/pubmed/?term=Wieczorowska-Tobis%20K%5BAuthor%5D&cauthor=true&cauthor_uid=21979452), [Wiecek A](https://www.ncbi.nlm.nih.gov/pubmed/?term=Wiecek%20A%5BAuthor%5D&cauthor=true&cauthor_uid=21979452), [Bartoszek A](https://www.ncbi.nlm.nih.gov/pubmed/?term=Bartoszek%20A%5BAuthor%5D&cauthor=true&cauthor_uid=21979452), [Dabrowski A](https://www.ncbi.nlm.nih.gov/pubmed/?term=Dabrowski%20A%5BAuthor%5D&cauthor=true&cauthor_uid=21979452) et al. Medical, psychological and socioeconomic aspects of aging in Poland: assumptions and objectives of the PolSenior project. Exp Gerontol. 2011; 46(12):1003-9 (research article)

**Portugal (n=1)**

National survey of the Portuguese elderly nutritional status.
Madeira T, [Peixoto-Plácido C](https://www.ncbi.nlm.nih.gov/pubmed/?term=Peixoto-Pl%C3%A1cido%20C%5BAuthor%5D&cauthor=true&cauthor_uid=27423703), [Goulão B](https://www.ncbi.nlm.nih.gov/pubmed/?term=Goul%C3%A3o%20B%5BAuthor%5D&cauthor=true&cauthor_uid=27423703), [Mendonça N](https://www.ncbi.nlm.nih.gov/pubmed/?term=Mendon%C3%A7a%20N%5BAuthor%5D&cauthor=true&cauthor_uid=27423703), [Alarcão V](https://www.ncbi.nlm.nih.gov/pubmed/?term=Alarc%C3%A3o%20V%5BAuthor%5D&cauthor=true&cauthor_uid=27423703), [Santos N](https://www.ncbi.nlm.nih.gov/pubmed/?term=Santos%20N%5BAuthor%5D&cauthor=true&cauthor_uid=27423703), [de Oliveira RM](https://www.ncbi.nlm.nih.gov/pubmed/?term=de%20Oliveira%20RM%5BAuthor%5D&cauthor=true&cauthor_uid=27423703), [Yngve A](https://www.ncbi.nlm.nih.gov/pubmed/?term=Yngve%20A%5BAuthor%5D&cauthor=true&cauthor_uid=27423703), [Bye A](https://www.ncbi.nlm.nih.gov/pubmed/?term=Bye%20A%5BAuthor%5D&cauthor=true&cauthor_uid=27423703), [Bergland A](https://www.ncbi.nlm.nih.gov/pubmed/?term=Bergland%20A%5BAuthor%5D&cauthor=true&cauthor_uid=27423703) et al. National survey of the Portuguese elderly nutritional status: study protocol. BMC Geriatr. 2016;16:139; doi: 10.1186/s12877-016-0299-x (research article)

**Spain (n=1)**

Longitudinal Study Aging in Spain (ELES). Human and Social Sciences Centre from the Spanish National Superior Council of Scientific Researches Council (CSIC), the Gerontology Institute Fundación Instituto Gerontológico MATIA (INGEMA).
http://161.111.47.116/drupal/en (study website)

**Sweden (n=2)**

The Swedish National study on Aging and Care in Kungsholmen (SNAC-K). Stockholm Gerontology Research Center, Aging Research Center (ARC), Karolinska Institutet.
http://www.snac-k.se (study website)

Swedish Living Conditions Surveys. Statistics Sweden.
https://www.scb.se/en/finding-statistics/statistics-by-subject-area/living-conditions/living-conditions/living-conditions-surveys-ulfsilc/produktrelaterat/more-information/more-about-the-swedish-living-conditions-surveys (study website)

**USA (n=2)**

University of Michigan Health and Retirement Survey (HRS).
http://hrsonline.isr.umich.edu. (study website)

National Health and Aging Trends Study (NHATS). Johns Hopkins University Bloomberg School of Public Health and University of Michigan.
https://www.nhats.org (study website)

1. Documents excluded because they did not refer to any pre-defined indicators (n=19)

**Australia (n=2)**

Older Australia at a glance. Australian Institute of Health and Welfare.
https://www.aihw.gov.au/reports/older-people/older-australia-at-a-glance/contents/summary (web report)

Productivity Commission. Caring for Older Australians: Overview, Report No. 53, Final Inquiry Report. Canberra; 2011.
https://www.pc.gov.au/inquiries/completed/aged-care/report/aged-care-overview-booklet.pdf (report)

**Austria (n=1)**

Gesundheit und Krankheit der älteren Generation in Österreich. Endbericht. Gesundheit Österreich GmbH, Bundesministerium für Gesundheit. https://broschuerenservice.sozialministerium.at/Home/Download?publicationId=539 (report)

**Canada (n=1)**

Government of Canada. Action for Seniors report.
https://www.canada.ca/en/employment-social-development/programs/seniors-action-report.html (web report)

**France (n=3)**

Les comportements de santé des 55-85 ans. Analyses du Baromètre santé 2010. Institut national de prévention et d’éducation pour la santé.
https://www.santepubliquefrance.fr/publications (report)

Vieillir en bonne santé. Santé publique France.
https://www.santepubliquefrance.fr/recherche/#search=Vieillir%20en%20bonne%20sant%C3%A9 (website)

Pour bien vieillir. Direction générale de la santé (DGS), la Direction des sports et la Direction générale de l'action sociale.
http://www.pourbienvieillir.fr (website)

**Germany (n=2)**

Gesundheit und Krankheit im Alter. Beiträge zur Gesundheitsberichterstattung des Bundes. Statistisches Bundesamt, Deutsches Zentrum für Altersfragen, Robert Koch-Institut. 2009. https://www.rki.de/DE/Content/Gesundheitsmonitoring/Gesundheitsberichterstattung/GBEDownloadsB/alter_gesundheit.pdf?__blob=publicationFile (report)

Gesundheit im Alter. Robert Koch-Institut, Gesundheitsmonitoring. https://www.rki.de/DE/Content/Gesundheitsmonitoring/Themen/Gesundheit_im_Alter/Ges_alter_node.html (website)

**Netherlands (n=1)**

Gezond ouder worden in Nederland. Rijksinstituut voor Volksgezondheid en Milieu (RIVM).
https://www.rivm.nl/bibliotheek/rapporten/270462001.pdf (report)

**Norway (n=2)**

Health and ageing in Norway - Public Health Report. Norwegian Institute of Public Health (NIPH).
https://www.fhi.no/en/op/hin/groups/health-and-ageing-in-norway (web report)

Healthcare Atlas for the Elderly in Norway. Northern Norway Regional Health Authority's Centre for Clinical Documentation and Evaluation (SKDE).
Balteskard L, Otterdal P, Steindal AH, Bakken T, Førde OH, Olsen F, Leivseth L, Uleberg B. Healthcare Atlas for the Elderly in Norway .An overview and analysis of publicly funded somatic health services for the population 75 years and older for the periode2013–2015
https://helseatlas.no/sites/default/files/healthcare-atlas-elderly.pdf (report)

**Scotland (n=2)**

Health and social care needs of older people in Scotland: an epidemiological assessment. Scottish Public Health Network (ScotPHN) 2013.
https://www.scotphn.net/wp-content/uploads/2015/10/Health-and-social-care-needs-of-older-people-in-Scotland-an-epidemiological-assessment.pdf (report)

Abayaratne D, Aresu M, Gharib W, Hirani V, Jones H, Mindell J, Roth M, Shelton N, Tabassum F.

The Scottish Health Survey: Topic Report: Older People's Health. Scottish Government 2011. https://www2.gov.scot/resource/doc/363507/0123373.pdf (report)

**Slovenia (n=1)**

Active and healthy ageing in Slovenia (AHA.Si), Communication and dissemination report, Version 1.0, 2016. National Institute of Public Health (NIJZ), Ministry of Health, Ministry of Labour, Family, Social Affairs and Equal Opportunities. http://www.staranje.si/sites/www.staranje.si/files/upload/images/aha.si_communication_report_v2.0_eng.pdf (report)

**Switzerland (n=2)**

Themenmonitoring „Gesundheit in der zweiten Lebenshälfte“. Arbeitsdokument des Obsan 11, April 2005. Schweizerisches Gesundheitsobservatorium. https://www.obsan.admin.ch/sites/default/files/publications/2015/arbeitsdokument-11-d.pdf (report)

Schweizerische Gesundheitsbefragung 2012. Die funktionale Gesundheit von älteren Menschen in Privathaushalten. Bundesamt für Statistik (BFS), 2014. https://www.bfs.admin.ch/bfs/de/home/statistiken/gesundheit/gesundheitszustand/alter.assetdetail.349311.html (report)

**Wales (n=2)**

Health Needs Assessment 2006: Older People. Version 2b. National Public Health Service for Wales. Health Information Analysis Team.
http://www.publichealthwales.wales.nhs.uk (report)

A Profile of the Health of Older People in Wales. National Public Health Service for Wales; 2004. http://www2.nphs.wales.nhs.uk:8080/hiatdocs.nsf/61c1e930f9121fd080256f2a004937ed/1cbe80f3742780518025726f0040a32b/$FILE/Older%20Persons%20-%20Chapter1Introduction.pdf (report)

1. Documents excluded because indicators relied on data from a single survey (n=3)

**Canada (n=1)**

Healthy Aging Indicators 2008/2009. Statistics Canada. https://www150.statcan.gc.ca/t1/tbl1/en/tv.action?pid=1310046601 (website)

**Turkey (n=1)**

Turkey Health Survey. Turkish Statistical Institute.
http://www.turkstat.gov.tr/Start.do (website)

**United States of America (n=1)**

The State of Aging and Health in America 2013. Centers for Disease Control and Prevention, US Dept of Health and Human Services; 2013.
https://www.cdc.gov/aging/pdf/State-Aging-Health-in-America-2013.pdf (report)

1. Documents excluded because indicators referred to a special aspect of health in older age (n=1)

**Canada (n=1)**

Age-Friendly Communities Evaluation Guide: Using Indicators to measure progress. Public Health Agency of Canada; 2015.
http://www.phac-aspc.gc.ca/seniors-aines/alt-formats/pdf/indicators-indicateurs-v2-eng.pdf (report)
